# Impact of the self-directed learning approach and attitude on online learning ineffectiveness: The mediating roles of internet cognitive fatigue and flow state

**DOI:** 10.3389/fpubh.2022.927454

**Published:** 2022-08-04

**Authors:** Mingming Shao, Jon-Chao Hong, Li Zhao

**Affiliations:** School of Education Science, Nanjing Normal University, Nanjing, China

**Keywords:** online learning, self-directed learning approach, self-directed learning attitude, internet cognitive fatigue, flow, learning ineffectiveness

## Abstract

Online learning has become an important learning approach in universities. However, since many students may have been exposed to online learning for the first time during this period of the COVID-19 pandemic, the quality factors of online learning and psychological distress of students need to be considered in the research on their learning. This paper discusses factors that influence the learning effect of university students in the online learning environment. A total of 377 university students participated in the survey. Structural equation modeling was used to verify the research hypotheses. The results show that the self-directed learning (SDL) approach and attitude can negatively predict students' Internet cognitive fatigue (ICF) and positively predict their Flow, whereas perceived learning ineffectiveness can be predicted by Internet cognitive fatigue positively and by Flow state negatively. The results can be a reference for online teachers to enhance students' online SDL attitude, and to discipline their SDL approach so as to promote online learning effectiveness.

## Introduction

Online learning has been widely adopted since 2020 (1). In order to achieve better online learning effectiveness, the realization and maintenance of online learning quality must be addressed (2). Moreover, psychological distress, such as attention and self-directed learning, has a great influence on online learning (3). Self-directed learning (SDL) involves the whole learning process from diagnosing learning needs, describing learning objectives, to evaluating learning outcomes by the learners themselves (4). Kim et al. (5) suggested that designing effective learner content to promote students' interaction is the most important work in maintaining their motivation for online learning (5). In these courses the quality of learner-content interaction may not be a predominant factor; rather, individual self-directed learning is more important (6). Thus, to explore the learning effectiveness of online learning, this study investigated learners' self-directed learning related to their achievement of the desired learning outcomes.

As Cinquin et al. (7) pointed out, it cannot be expected that all students will find online tools beneficial, as students differ in their learning preferences and styles (7). In particular, some online courses may result in impairments of cognitive function (attention, memory, etc.). On the other hand, when navigating online learning environments, some learners may experience a state of flow (8). While in a flow state, learners concentrate on the activity being performed and lose awareness of other environmental stimuli unrelated to their learning (9). Some studies have indicated that students who experience Internet cognitive fatigue may not enjoy online learning (10). Flow experience has been found to effectively enhance online learning, for example, learning English as a second language (11). However, few studies have discussed how these two mental states interact during online learning. Thus, the present study also explored learners' Internet cognitive fatigue and flow state while they were involved in the online learning process.

Learning outcome is one of the measurements to assess how effective a learning platform is. The perception of the learning effectiveness of online learning is dependent upon whether the desired outcomes are achieved (12). The “dark” aspect of psychology indicates that young people are inclined to view bias in the social world through the external manifestations of lower grades (13). For example, adolescent students tend to make negative evaluations of social norms (14). Thus, in this study, learning performance was replaced by learning ineffectiveness so as to better enable participants to make self-evaluations of their own learning performance perceptions (15). Moreover, learning effectiveness perceived in different contexts is important to understand, as different learning interventions might influence the effectiveness differently. Thus, this study established a structural equation model to explore the influence of two aspects of self-directed learning (approach and attitude) on the different performance of attention (cognitive fatigue and flow state), and the role of these four factors on the effect of online learning performance. According to the research results, people can train learners' self-directed learning approach and give targeted guidance to their self-directed learning attitudes, so as to promote concentration and avoid cognitive fatigue, effectively improving the online learning performance in the future.

## Theoretical background

### Self-directed learning approach and attitude

Tough (16) first proposed the concept of “self-directed learning” (SDL) as a way of learning (16). Knowles (4) defined it as an approach whereby learners diagnose their own learning needs, clearly describe their learning objectives, look for learning resources, choose and implement suitable learning strategies, and evaluate their learning outcomes, all without others' help (4). Caffarella (17) described SDL as an attitude toward autonomous learning (17). Taken together, SDL comprises two orientations: attitude orientation and approach orientation. In attitude orientation, SDL is considered to be a personal trait of the learner. As well as having different attitude orientations, learners also have different degrees of autonomy. In the approach orientation, the emphasis is placed on learners' learning activities such as their planning and implementation of learning strategies in and after class (18). Moreover, attitudes and actions (approaches) can generate context-related learning effectiveness (19). University students' SDL when taking online courses, including the self-directed learning approach and self-directed learning attitudes, was introduced into this study.

The trait activation theory (TAT) explicates how work situations comprising shared challenge and hindrance stressors can be relevant for the expression of online learning (20). TAT highlights important interactions between person and situation variables. In this context, self-directed learning in online learning was proposed. Song and Hill (21) began to focus on self-directed learning in an online learning environment, and built a SDL model in an online context that combined SDL with personal attributes and learning processes, indicating the impact of environmental factors on SDL (21). Kim et al. (22) took a closer look at the application of self-directed learning in the field of online learning (22). They found that SDL could help students who studied online to develop the characteristics of a personalized system, and to improve their ability to manage overall learning activities and monitor their own performance, which could in turn help them to better adapt to online learning. As the expectation of SDL is that individual learners assume responsibility for their own online learning depending on their unique needs and individual goals (23), the roles that the two types of SDL play in online learning have not been extensively discussed. Thus, this study explored university students' two types of SDL, SDL-approach and SDL-attitude, while they learned online.

### Internet cognitive fatigue and flow state

The inability to maintain attention is central to the concept and operational definition of cognitive fatigue (24), which has been defined as an executive failure during time spent performing tasks. It involves neglectfulness, loss of memory, distracted attention, as well as a lack of concentration (25). It evokes mind wandering, which may also interfere with other mental processes (26). Cognitive appraisal has been defined as “an evaluative process that determines why and to what extent a particular transaction or series of transactions between the person and the environment is stressful” (27). In this study, Internet cognitive fatigue (ICF) that is the result of using LINE is referred to as LINE cognitive fatigue (28). ICF may affect students' learning performance, such as by causing distraction and reducing focus, creating a heavy mental load, and causing problems with Internet usage that recurrently influence learning performance (29). In contrast to cognitive fatigue, Csikszentmihalyi (30) introduced the flow state. Flow is defined as a state in which individuals are so deeply engaged in the current activity that they do not pay attention to other activities or the passage of time (30). This state was defined as a holistic experience in which individuals perceive themselves as being totally involved (31). When they are in a flow state, they are absorbed in the activity they are performing, and the focus of their awareness is targeted. Their minds become more unwandered, and they perceive themselves as being able to control their environment (32, 33).

Many of the studies on mental state while using the Internet have used the flow concept to address online navigation phenomena, but they have produced mixed evidence regarding the efficacy of such online learning (34). Thus, this study examined two types of mental state, namely ICF and Flow, in order to clarify some of the reported ambiguities regarding the conceptualization and operationalization of the effectiveness of online learning.

### Learning ineffectiveness

In the research on online learning, some studies have focused on the hurdles that impede the effective delivery of online courses; for example, in massive and emergency online platforms (35, 36), factors such as the unpreparedness of most administrators, staff members, and students are hurdles (29). Of particular note is students' desire for learning effectiveness (37). Existing research has shown that young adults try to associate self-perception biases with behavioral outcomes and look down on external attributes (38). Hong et al. (15) used learning ineffectiveness to explore online learning effect (15). Accordingly, the present study considered the role of online learning ineffectiveness related to remote learning.

### Literature summary and research significance

Although there has been some research on self-directed learning and attention in academia (24), and on the relationship between them (39), few scholars have explored the significance of self-directed learning by dividing it into learning approach and learning attitude. Some learners have mastered the method of self-directed learning but are not willing to carry it out, while others want to carry it out but do not know how to learn scientifically and effectively. Both of these situations can lead to the failure of self-directed learning, which can be linked with two different forms of attention: cognitive fatigue and concentration. It is also innovative to link these two conditions to two different forms of attention: Internet cognitive fatigue and flow state. It would be interesting to see whether these two different conditions promote concentration or divergence.

In addition, since teenagers tend to have more positive self-perceptions, and what they perceive may not be the same as what they perform, the existing research resulting to promote learning performance may not be suitable (38). Therefore, it is also of great significance to explore whether the conversion of the scale into learning ineffectiveness is different from the positive learning performance results. At the same time, the learning environment of online learning is different from that of traditional learning environments. The situation of online learning changes the self-directed learning and attention of students, which also has an impact on learning effectiveness.

## Research model and hypotheses

### Hypotheses

While students are navigating online learning environments, they may perceive challenges that link to opportunities for action. When they are in a state of flow, they also engage in and focus on the activity they are performing; they may focus or lose concentration on any environmental change. This is considered desirable insofar as it changes their mental state so that they realize that the challenges they face are in balance with their learning attitude and approaches (40). When students are engaged in online learning, their self-directed learning attitude may affect their ICF and flow states (8). However, few researchers have discussed how the self-directed learning approach and attitude affect the ICF and Flow in the particular context of online learning; thus, the following hypotheses were proposed:

**H1** SDL-approach is negatively related to ICF.
**H2** SDL-approach is positively related to Flow.
**H3** SDL-attitude is negatively related to ICF.
**H4** SDL-attitude is positively related to Flow

Online courses have increased the accessibility of learning, but students' ability to concentrate is an important factor in measuring the quality of their online learning (41). Attention guidance can facilitate students' constructive use of instructional materials when they engage in online learning conversations (42). On the other hand, students often carry out online learning in situations where they are easily distracted (43). Many students have reported that they find it difficult to pay attention (44), and previous researchers have aimed to identify when mind wandering occurs. However, few have discussed how students' ICF and Flow affect their perceptions of learning ineffectiveness in the particular context of online learning; thus the following hypotheses were proposed:

**H5** ICF is positively related to students' learning ineffectiveness.
**H6** Flow is negatively related to students' learning ineffectiveness in online learning.

### Research model

**Figure 1:**
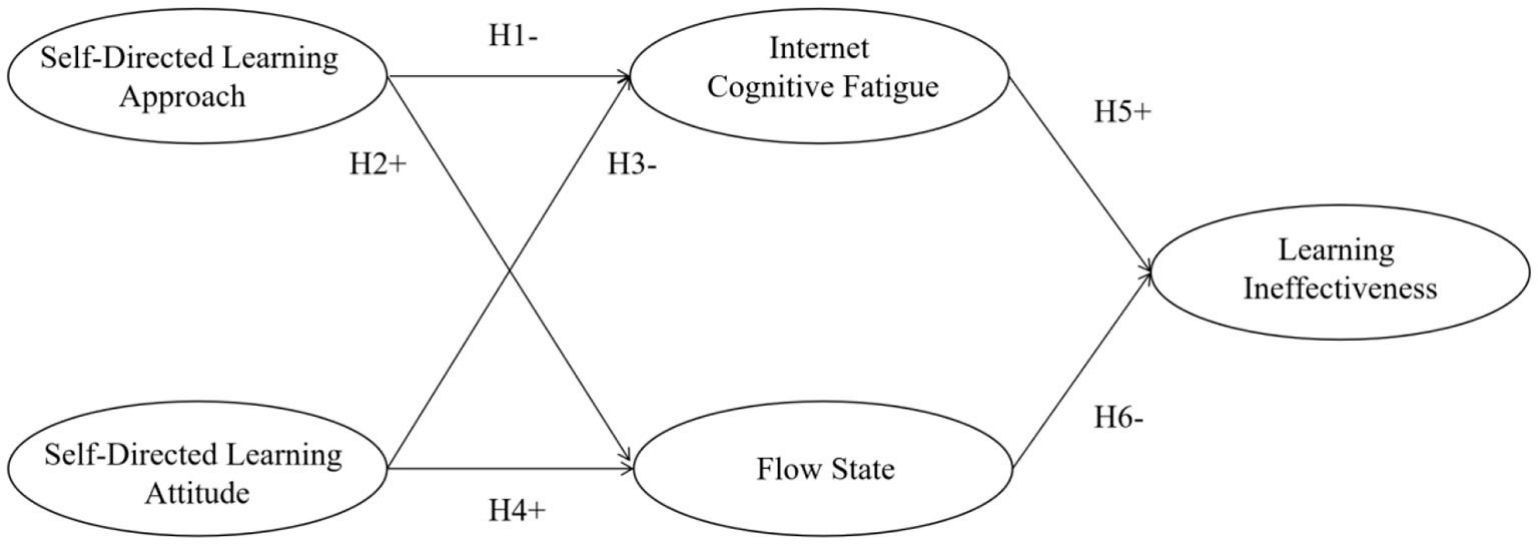
Research model.

The cognitive appraisal theory (CAT) categorizes personal traits in terms of positive or negative valence, which can trigger different psychological states (45). Moreover, according to the environmental psychology theory (46), it is assumed that the set of physical and tangible cues in an environment affects users' emotional states and behaviors. In the COVID-19 environment of online learning, the self-directed learning approach and attitude serve as mental state antecedents, and learning ineffectiveness is a mental state consequence. As a result, the present study proposed a model to identify individual traits that are subject to environmental factors which shape an individual's vulnerability to COVID-19 as stressor-related online learning problem. Therefore, the research model was conceptualized as shown in Figure 1.

## Method

### Participants and procedure

A survey with a questionnaire was administered to university students with online learning experience in Jiangsu province, China. The questionnaire was uploaded to an online tool called *Questionnaire Star* (www.wjx.com). A web site, valid for participants to access for one month, was generated and the link was randomly sent to 50 university students in Jiangsu. Participants were then invited to share the link with their classmates. A total of 384 questionnaires were collected. After deleting those questionnaires with unanswered items, the same answer to all items, and less than 2-minute answering time, 377 valid questionnaires remained, giving an effective response rate of 98%.

### Instruments

The questionnaire was designed by adapting from previous studies. Two professors majoring in psychology and three in education checked and revised the accuracy of the item statements, using the forward-backward translation approach to obtain the face validity of the questionnaire. A 5-point Likert scale was designed, with 1 for strongly disagree, and 5 for strongly agree. After data collection, the reliability and validity of the questionnaire items and constructs were tested. The questionnaire consists of three parts. The first part is the introduction of the survey and the explanation of the data collected only for the participants with online learning experience. The second part is the investigation of the basic information of the subjects. The third part is the main part of the questionnaire, including the potential variables of SDL-approach, SDL-attitude, Internet cognitive fatigue, and Flow state.

#### SDL measurement

Self-directed learning can help to understand an individual's attitude toward online learning and provide further insight into how an individual can use learning methods in an online environment (23). This study adopted the scale of Sun (47), which divides self-directed learning into two aspects: approach and attitude. In the original scale, there were five items for self-directed learning approach and five for self-directed learning attitudes (47). The descriptions of these 10 items were adapted according to the circumstances of this study.

#### SDL-approach measurement

Self-directed learning is the foundation of all learning, whether formal or informal, and the effectiveness of learning is related to individual motivation. All people are capable of self-directed learning, but their development level varies due to individual methods (48). Accordingly, five items were adapted related to how we should achieve self-regulation in learning. Exemplary items include: I can make my own study plan effectively, and When I encounter problems with the use of the online learning system, I will find the best solution by myself.

#### SDL-attitude measurement

Crook (49) explained that autonomous learners are active and take the initiative in learning, rather than passively waiting to be taught (49). As people take more responsibility for their own lives and benefit from self-discipline in the learning process, self-directed learning attitude refers to whether they have a strong willingness to learn independently. Accordingly, five items were adapted in this study, for example: When a new concept or thing comes along, I like to explore it myself, and When I come across something I don't understand, I like to try to find a solution on my own.

#### Internet cognitive fatigue measurement

This measure referred to the scale of Schwid (50), where cognitive fatigue is thought to be a cognitive decline on tests that require sustained attention. Hong et al. (10) and Hwang et al. (28) mentioned the cognitive decline related to interaction with internet information (10, 28). There were seven items in the original questionnaire pool to explore participants' perceptions of cognitive fatigue. Since the participants of the original questionnaire were those with a steady job, the project description was revised in this study and two questions unsuitable for student participants were deleted. Accordingly, five items were adapted. Examples include: When I study online, I am distracted by the interaction of different avatars, and I cannot quickly grasp what others are saying, and When studying online, if the teacher talks too much at one time, I can't understand.

#### Flow measurement

When people are in a state of flow, they become absorbed in the activity they are performing, the focus of their awareness becomes narrower, they are less conscious of themselves, and they feel that they have control of their environment (31). Based on the understanding of flow state in existing studies, eight items were self-compiled for this study; examples are: When studying online, I can concentrate on class for a long time, and When I study online, I won't listen and think about other things.

#### Perceived ineffectiveness of online learning measurement

Online learning ineffectiveness was introduced by Hong et al. (15). Considering the “dark” psychology of young adults, Hong et al. (15) used ineffectiveness rather than effectiveness when designing items for students' online learning performance (15). There were nine items in the original questionnaire, but one item with low reliability was deleted in this study. Accordingly, eight items were adapted; examples are: Since learning online, the quality of my homework has deteriorated, and Since online study began, my ability to observe and find problems has become weaker.

## Results

First-order CFA was first applied to determine the reliability of the tool and to delete unreasonable questionnaire items. The reliability and validity of variables were tested to determine the credibility of the research instrument. Finally, structural equation modeling (SEM) was used to verify the hypothetical structural model. In this study, SPSS 24.0 was used for descriptive statistics and reliability and validity analysis, and AMOS 24.0 was used for CFA and path analysis of the structural model.

### Participant information

Of the respondents, 29.2% were males and 70.8% were females, 40.3% were freshmen, 38.7% were sophomores, and 21.0% were juniors (no seniors were recruited because most university senior students were in internships and were not participating in school courses at the time). As for their online learning time, about 13.5% spent 1–2 h per week, 51.2% spent 2–4 h per week, 24.4% spent 4–6 h, and only 10.9% students had more than 6 h per week of online learning. Regarding the number of online courses, 17.0% had 1–3 courses, 76.7% had 4–6, 5.6% had 7–9, and only 0.8% had more than 10 online courses.

### Item analysis

The original questionnaire had 31 items in total, including SDL-approach, SDL-attitude, ICF, Flow, and learning ineffectiveness. When a sample is used in first-order confirmatory factor analysis (CFA), if the factor loading is less than 0.5, the items should be deleted (51). Moreover, the highest residual values of items in each construct should be deleted until the threshold value met the first-order CFA requirements (51). The value of GFI was 0.919; NFI was 0.943; CFI was 0.972; RMSEA was 0.049; and χ^2^/*df* was 1.907. Accordingly, the following questionnaire items were retained: self-directed learning approach (3 items), self-directed learning attitude (4 items), ICF (4 items), Flow (4 items) and learning ineffectiveness (6 items), giving a total of 21 items.

### Construct reliability and validity analysis

SPSS 24.0 was used to analyze the reliability and validity of the questionnaire. Cronbach's alpha was adopted for the internal consistency analysis. Table 1 shows that the Cronbach's alpha of all constructs was higher than 0.8. The composite reliability (CR) is for measuring the external consistency of constructs. In this study, CR values ranged from 0.82 to 0.95, indicating acceptable validity (51).

**Table 1:** Dimension reliability and validity analysis.

**Variable**	**Measure item**	**M**	**SD**	**FL**	**CR**	**AVE**	**Cronbach's Alpha**
Self-directed learning approach	SDL-approach 1	3.45	0.791	0.774	0.8176	0.5991	0.819
	SDL-approach 2	3.62	0.752	0.770			
	SDL-approach 3	3.62	0.807	0.778			
Self-directed learning attitude	SDL-attitude 1	3.69	0.875	0.787	0.8888	0.6669	0.889
	SDL-attitude 2	3.86	0.911	0.781			
	SDL-attitude 3	3.64	0.839	0.852			
	SDL-attitude 5	3.69	0.822	0.844			
Internet Cognitive Fatigue	ICF1	2.67	0.983	0.858	0.8871	0.6628	0.893
	ICF2	2.40	0.873	0.800			
	ICF3	2.32	0.841	0.796			
	ICF5	2.80	1.056	0.801			
Flow	Flow 4	3.90	0.769	0.793	0.8871	0.6629	0.897
	Flow 5	3.98	0.711	0.795			
	Flow 6	3.89	0.733	0.857			
	Flow 7	4.03	0.710	0.810			
Learning ineffectiveness	LI1	2.62	1.043	0.729	0.9495	0.7593	0.950
	LI2	2.57	1.047	0.887			
	LI3	2.56	1.080	0.942			
	LI4	2.54	1.118	0.896			
	LI5	2.54	1.108	0.922			
	LI6	2.62	1.066	0.835			

*M, Mean; SD, Standard Deviation; FL, Factor Loading; CR, Composite Reliability; AVE, Average Variance Extracted*.

According to Fornell and Larcker's (52) study of convergent validity, the higher the convergent validity, the higher the factor loading (FL) (52). According to the previous research, a FL above 0.7 is considered a good value. The AVE (Average Variance Extracted) value should exceed 0.5, indicating that the construct has the effect of convergence. Table 1 shows that all values of FL and AVE are above 0.5, indicating that the questionnaire had a high degree of validity.

When performing construct discriminant validity analysis (as shown in Table 2), we must first obtain the square root of AVE for each dimension, and it should exceed the absolute value of the Pearson correlation coefficient between the two dimensions (53). In the current study, the analysis showed that the square root of AVE value of all variables exceeded the absolute value of the correlation coefficient between variables, thus indicating that the measurement model had good discriminative validity (53).

**Table 2:** Dimension discriminant validity analysis.

**Construct**	**1**	**2**	**3**	**4**	**5**
1. SDL-approach	**0.774**				
2. SDL-attitude	0.388	**0.817**			
3. ICF	0.469	0.422	**0.814**		
4. Flow	0.527	0.435	0.405	**0.814**	
5. Learning ineffectiveness	0.193	0.186	0.307	0.303	**0.871**

*The diagonal elements (bold) are the square roots of AVE and the off-diagonal elements are values of the inter-construct correlations*.

### Hypothesis testing and path analysis

In this study, the absolute fit index and relative fit index were used to evaluate the degree of fit of the model. The value of GFI is 0.906 which is more than 0.9 and <1.0 (54). NFI and CFI should both be > 0.9 (44). The value of NFI is 0.932 and CFI is 0.961. RMSEA should be <0.1 (44), and here it is 0.057. From the perspective of the model indexes as Table 3 shows, the χ^2^/*df* , RMSEA, GFI, CFI, NFI, and IFI all fell within the acceptable ranges, illustrating that the model of this study fits the data well.

**Table 3:** Model fitting analysis.

**Fitting index**	**Threshold**	**Values**	**Results**
Chi-square/*df*	<3	2.234	Supported
RMSEA	<0.08	0.057	Supported
Goodness-of-fit index (GFI)	>0.8	0.906	Supported
Adjusted fitness index (AGFI)	>0.8	0.882	Supported
Normed fitness index (NFI)	>0.9	0.932	Supported
Non-normalized fitness index (NNTI/TFI)	>0.9	0.955	Supported
Comparative fitness index (CFI)	>0.9	0.961	Supported
Incremental fitness index (IFI)	>0.9	0.961	Supported
Relative fitness index (RFI)	>0.9	0.922	Supported

The hypotheses of the research model were tested by path analysis of the relationship among variables. Table 4 shows that the significance of the five hypotheses proposed in this study was verified. There are significant states among the hypotheses. All of the *p*-values are <0.001. The SDL-Approach and SDL-Attitude have a direct negative association with ICF (β = −0.592, *t* = −7.704^***^; β = −0.366, *t* = −6.305^***^), while the SDL and SDLA have a direct positive association with Flow (β = 0.514, *t* = 8.806^***^; β = 0.264, *t* = 6.308^***^). Moreover, ICF has a direct positive association with LI (β = 0.202, *t* = 3.728^***^), and Flow has a direct negative association with LI (β = −0.273, t = −3.732^***^).

**Table 4:** Path coefficient analysis.

**Hypothesis**	**Causal factors**	**Standardized** **coefficient (β)**	**S.E**.	***t***	***p***	**Result**
H1	SDL-approach → ICF	−.592	.082077	−7.194704	*p* <0.001	supported
H2	SDL-approach → Flow	.514	.058	8.806	*p* <0.001	supported
H3	SDL-attitude → ICF	−.366	.058	−6.305	*p* <0.001	supported
H4	SDL-attitude → Flow	.264	.042	6.308	*p* <0.001	supported
H5	ICF → LI	.202	.054	3.728	*p* <0.001	supported
H6	Flow → LI	−.273	.073	−3.732	*p* <0.001	supported

**Figure 2:**
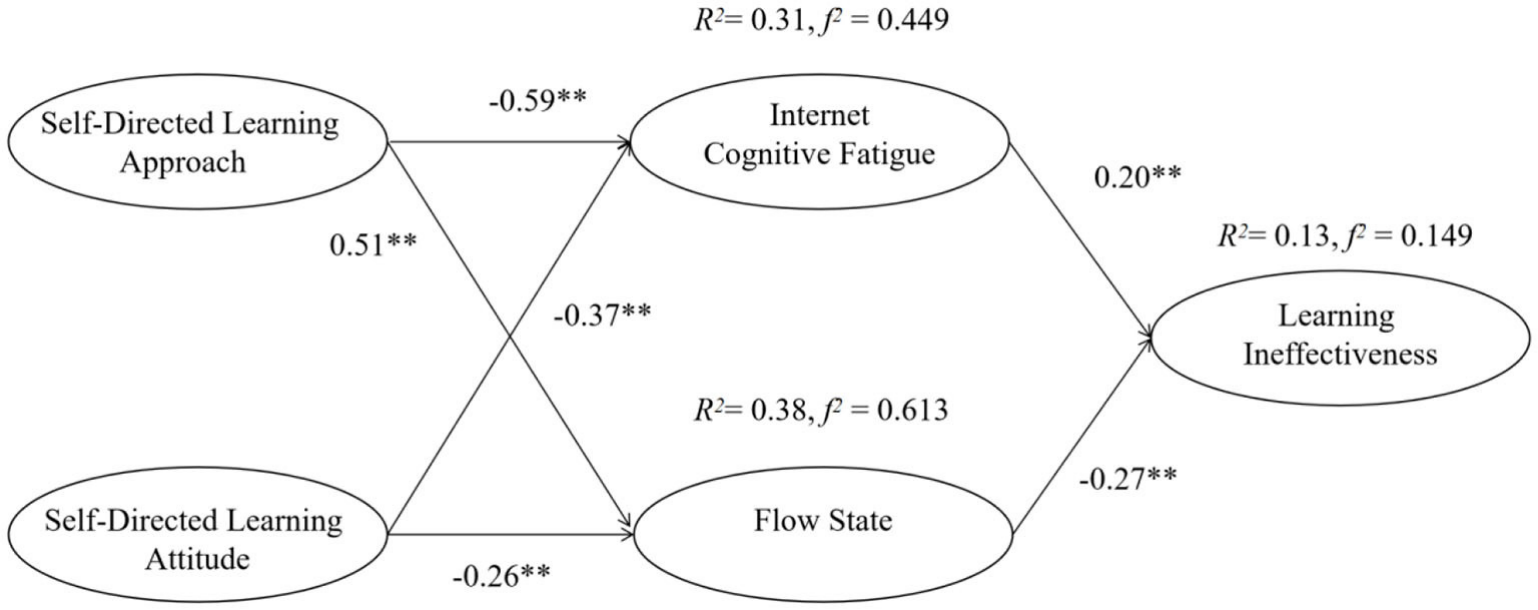
The verification of the research model. **p < 0.01.

The determination coefficient *R*^2^ quantifies the variance ratio interpreted by the statistical model. It is an important statistic for summarizing biological benefits. When *R*^2^ values are <0.6, we consider that 0.3–0.6 is medium, and <0.3 is low (55). In addition, the model effect size (*f* ^2^) was proposed by Cohen (56) to enable researchers to move from simply recognizing statistical significance to providing a more general quantifiable description of the size of the effect (57). *f* ^2^ values > 0.8 can be considered large. When *f* ^2^ is between 0.2 and 0.8, it can be considered medium, and when it is <0.2, it can be considered small. In this study, the explanatory power of SDL and SDLA on ICF is 31% (*R*^2^ = 0.31, *f* ^2^ = 0.449), and Flow is 38% (*R*^2^ = 0.38, *f* ^2^ = 0.613). The explanatory variance of CF and Flow on LI is 13.0% (*R*^2^ = 0.13, *f* ^2^ = 0.149). The six variables in this study are therefore shown to have good predictive power (44). However, in order to improve the degree of fit of the model, adjustments to the model were made, as shown in Figure 2.

## Discussion

In the existing studies, taking COVID-19 as source of stress, there were many studies on the theoretical literature describing how COVID-19 may affect online learning, for example, similarities and differences between online learning and face-to-face learning (58–60). However, the empirical literature related to the two types of self-directed learning that affect individual mental state and learning effectiveness is limited. Thus, the present study explored the correlates between SDL-approach, SDL-attitude, Internet cognitive fatigue, flow experience, and perceived online learning ineffectiveness. After statistical analysis with item suitability, construct reliability and validity, structural equation modeling was applied to test the hypotheses. The results of this study are discussed as follows.

According to the TAT, individual traits are latent potentials residing in the individual attitude and approach; what triggers mental state is critical for understanding how the two types of SDL affect Internet cognitive fatigue and flow experience in this study. Self-directed learning can be defined as the mode of learning in which students who establish their own study goals and strategies are accountable for outcomes. It is essential to learn by oneself under the threat of COVID-19 (61). According to environmental psychology theory, environmental change may activate or deactivate individual mental activities, and self-directed learning should be prioritized with online learning (61).

Moreover, when students are in a state of flow, they are engaged in and focused on performing the activity, and they may focus or lose concentration as a result of any environmental change (30). ICF may affect students' online learning performance, such as by causing distraction and reduced focus, heavy mental load, and problems with Internet usage that recurrently influence their learning performance (29). Because online learning, which includes either watching video lectures or attending real-time video class meetings, is relatively unrestricted in terms of time and space, individuals can proactively steer the learning environment and accommodate SDL (61). Moreover, as the expectation of SDL is that individuals will assume responsibility for their own online learning depending on their unique needs and individual goals (23), SDL attitude and approach can balance the challenge of online learning and result in a change in mental state (40). How two types of SDL affect two types of mental state in online learning was explored in this study. The results revealed that the SDL approach is negatively related to ICF but positively related to Flow, whereas SDL attitude is negatively related to ICF but positively related to Flow.

In line with TAT, shared challenge stressors may overwhelm groups to achieve desired work outcomes. On the other hand, taking COVID-19 as a hindrance stressor will inhibit psychometric responses to self-evaluation. Mental states can facilitate or inhibit students' constructive use of instructional materials when they engage in online learning conversations (42, 43). Some studies have reported that paying attention is more difficult when mind wandering occurs (44). However, to explore how students' ICF and Flow affect their perceptions of learning ineffectiveness in the particular context of online learning, the present study found that ICF was positively related to learning ineffectiveness, suggesting that the higher the learner's ICF, the lower their learning performance would be. Thus, H5 is true. Flow is the opposite of ICF, so the higher a learner's level of concentration, the higher their learning performance is; H6 is thus also proved.

## Conclusion

According to cognitive appraisal theory, environmental psychology theory, and trait activation theory, this study puts forward that the four factors of self-directed learning approach and self-directed learning attitude, ICF, and Flow will directly or indirectly affect the quality of university students' online learning. Through path analysis for the model, it was found that the self-directed learning approach and self-directed learning attitude can predict two types of mental state: negatively to ICF and positively to Flow. Moreover, ICF can negatively predict and Flow can positively predict learning ineffectiveness. Therefore, the influence of psychological distress on online learning should be taken seriously.

### Implications

The results show that self-directed learning can predict mental state, and has a direct or indirect impact on the ineffectiveness of online learning, confirming that the four factors have a significant influence on learning ineffectiveness. That is similar to the findings of other studies. When conducting online learning for university students, more attention should be paid to the cultivation of students' SDL awareness, carrying out relevant lectures, strengthening the training of their SDL approaches and paying attention to guiding their attitude toward SDL in online learning.

### Limitations

There are some limitations of the study that should be noted. First, the snowball sampling method was used to connect with a limited population of university students in one area. Future studies should involve a greater number of participants from a variety of different areas. Second, this study adopts the cross-sectional survey results of a node at a certain time. In the future, more longitudinal data at different time points should be collected, which will increase the objectivity and stability of the conclusions and increase the rigor of the study. Last but not least, the present study focused on cognitive appraisal evaluation under the stress of COVID-19 and explored the correlates between individual trait and mental state of online learning reflected in learning effectiveness, without considering the comparison of types of online learning and online learning effectiveness. Future studies may compare different online approaches to examine participants' cognitive and effective issues.

## Data availability statement

The original contributions presented in the study are included in the article/supplementary material, further inquiries can be directed to the corresponding author.

## Ethics statement

Ethical review and approval were not required for the study on human participants in accordance with the local legislation and institutional requirements. The survey data participants provided were anonymous and would not be of any commercial use.

## Author contributions

All authors contributed equally to the conception of the idea, implementing and analyzing the experimental results, writing the manuscript, and reading and approving the final manuscript.

## Funding

This study was funded by National Social Science Foundation (No. BCA200093).

## Conflict of interest

The authors declare that the research was conducted in the absence of any commercial or financial relationships that could be construed as a potential conflict of interest.

## Publisher's note

All claims expressed in this article are solely those of the authors and do not necessarily represent those of their affiliated organizations, or those of the publisher, the editors and the reviewers. Any product that may be evaluated in this article, or claim that may be made by its manufacturer, is not guaranteed or endorsed by the publisher.
